# “Our lives matter”: a qualitative examination of the impact of COVID-19 shelter-in-place orders on resource security and mental and physical health of Black and Latino sexual minority men in California and New York

**DOI:** 10.1080/29944694.2025.2530962

**Published:** 2025-07-14

**Authors:** Orlando O. Harris, Joseph Egbunikeokye, Whitney D. Bagby, Abel Rivas, Natalie Wilson, Jerry John Ouner, Jose I. Gutierrez, Glenn-Milo Santos, Mitchell Wharton

**Affiliations:** a Department of Community Health Systems, School of Nursing, University of California, San Francisco, California, USA; b Department of Family Health Care Nursing, University of California, San Francisco, California, USA; c Us Helping Us, People Into Living, Inc, Washington, District of Columbia, USA

**Keywords:** COVID-19, mental health, sexual minority men, shelter-in-Place, resource insecurity

## Abstract

The COVID-19 and HIV pandemics have disproportionately affected Black and Latino sexual minority men (BLSMM) in the United States, with them having the highest burden of disease. Despite this disparity, few studies have examined the intersection of COVID-19 mitigation measures, access to healthcare, and other social determinants during the COVID-19 pandemic among BLSMM. To fill this gap in the literature, the purpose of this study is to explore the impact of the pandemic on mental and physical health, access to healthcare, and resource security among BLSMM. Using an interpretive phenomenological approach, we conducted individual interviews with 41 participants between August 2021 and December 2022. Interviews were recorded, transcribed, and analyzed using thematic content analysis. Participants ranged in age from 19–65 years. The majority described their gender as male (93%). Black participants comprised the majority of the sample (73%), with 25% identified as Latino. Participants’ narratives indicated a sense of hopelessness, despair, anxiety, and depression—all amplified by social isolation experienced from COVID-19 shelter-in-place (SIP) orders. Fear of contracting COVID-19 disrupted engagement in healthcare. Resource security was exacerbated by unemployment, resulting in food and housing insecurity and reliance on government assistance programs. These findings highlight the pandemic’s impact on participants’ mental and physical health, access to healthcare, and resource security. Our findings suggest a more nuanced and culturally tailored public health response approach, expanding telemedicine to increase access to healthcare, and other governmental policy changes to increase access to affordable housing and food, regardless of immigration status.

## Introduction

The novel coronavirus (SARS-CoV-2 [COVID-19]), the cause of an acute respiratory syndrome, was classified as a global pandemic in March 2020 (Berry et al., 2021; Harris et al., 2023; Yancy, 2020). Since its onset, COVID-19 remains a serious health threat worldwide, with over 776.2 million confirmed cases and over 7 million deaths as of September 15, 2024 (Centers for Disease Control and Prevention, 2024; Dong et al., 2020; Kaiser Family Foundation, 2024). In the United States (US), high COVID-19 morbidity and mortality have been documented among people with chronic health conditions, the elderly, uninsured individuals, sexual minority men (SMM), and racial and ethnic communities, especially those from low socioeconomic backgrounds (Grekousis et al., 2022; Harris et al., 2020; Selden & Berdahl, 2020). National efforts to reduce the spread of COVID-19 have led to unprecedented disruptions across all sectors, with significant social, economic, and healthcare impacts, including access to social events and interruptions in HIV prevention and treatment services (Couch et al., 2020; Dotson et al., 2022; Holloway et al., 2021; Mohr et al., 2021; Molitor et al., 2021). Therefore, disparities in health and social inequalities among marginalized groups, such as racial, ethnic, sexual, and gender minorities have also worsened (Baggett & Gaeta, 2021; Drake et al., 2021; Santos et al., 2022).

In March of 2020, at the beginning of the global COVID-19 pandemic, the Centers for Disease Control (CDC) issued shelter-in-place (SIP) orders, social distancing, and other prevention guidelines to state and local health departments in an effort to curb the spread of COVID-19 (Bowleg, 2020; Harris et al., 2020; Lodge & Kuchukhidze, 2020). While the majority of states across the country implemented those guidelines, there were state-level differences across the country (Feyman et al., 2020). As a result, the effectiveness and equitable application of SIP orders varied by state, creating racial disparities in COVID-19 risk in some communities (Block et al., 2020; Feyman et al., 2020). Regardless of jurisdiction, SIP orders contributed to social and physical isolation that increased loneliness, depression, anxiety, substance use, and other hazardous sexual behaviors (Carriedo et al., 2020; Gabster et al., 2022; Levy et al., 2022). SIP orders also created an environment where many individuals were sharing spaces for long extended periods of time, including those who live in multigenerational housing, increasing the likelihood of domestic violence and other social problems (Evans et al., 2021; Leslie & Wilson, 2020). Black/African Americans and Latinx people who are more likely to live in multigenerational housing experienced significant challenges as a result of SIP orders (Selden & Berdahl, 2020; Webb Hooper et al., 2020). In addition, BLSMM who rely on socialization with their peers through LGBTQ-oriented social spaces and gyms were more likely to report maladaptive behaviors due to SIP (Gauthier et al., 2021; Holloway et al., 2021; Tai et al., 2021).

In the first year of the pandemic, the national unemployment rate rose to over 14% among adults, while it was slightly higher among Black/African Americans (17%) and Hispanics (19%), amplifying preexisting social and financial disparities (Brooks et al., 2022; Couch et al., 2020; Hagen et al., 2022). Black/African American and Latino individuals are more likely to be considered essential workers or work in industries with limited social distancing precautions or no work-from-home options, increasing their risk of exposure to COVID-19 (Berry et al., 2021; Drake et al., 2021). In addition, due to persistent unemployment, food and housing insecurity, and lack of access to healthcare increased among this population (Dsouza et al., 2024; Molitor et al., 2021). Higher rates of unemployment have also been associated with increased mental health symptoms (Nagata et al., 2021; Parra et al., 2023; Selden & Berdahl, 2020). BLSMM experienced higher rates of unemployment due to the pandemic and SIP orders, which resulted in a lack of access to HIV prevention and treatment services (Evans et al., 2021; Lodge & Kuchukhidze, 2020; Rogers et al., 2020). Moreover, undocumented Black and Latino individuals who do not qualify for social programs or other governmental assistance experienced worsened disparities in health due to unemployment and lack of access to employer-based health insurance during the COVID-19 pandemic (Bhuiyan et al., 2021; Clark et al., 2020; Flores Morales & Farago, 2021).

SIP orders negatively impacted the mental and physical health of people across the country (Brooks et al., 2022; O’Brien et al., 2021). Many of the mental health challenges that arise due to the pandemic resulted in increased rates of depression, sadness, and anxiety (Fish et al., 2020; Holloway et al., 2021; Lea et al., 2023). Additionally, many of these challenges occurred at the height of the pandemic when access to mental health services was either paused or offered much later as teletherapy through an electronic web conferencing medium on a computer or cellphone (Fish et al., 2020; O’Brien et al., 2021). While considered to be an alternative to in-person therapy sessions, teletherapy poses some challenges with privacy in the context of multigenerational households, specifically in low-income communities (Elser et al., 2021; O’Brien et al., 2021; Parra et al., 2023). Racial disparities in terms of COVID-19, SIP orders, and mental health symptoms were also observed across the country, with Black and Latinx individuals experiencing disproportionate burdens (Brooks et al., 2022; Grumbach et al., 2021). There is some evidence that the pandemic has resulted in higher rates of substance use, depression, and anxiety among SMM compared to their heterosexual counterparts (Fish et al., 2020; Holloway et al., 2021; O’Brien et al., 2021). However, little is known about how the pandemic, along with the intersection of race, sexual orientation, and COVID-19 mitigation measures impacted the mental health and overall well-being of BLSMM, a significant gap this study hopes to fill.

Despite the end of the global and national COVID-19 pandemic and public health emergency restrictions, BLSMM continues to experience health and economic disparities, both of which are considered social determinants of health (Berry et al., 2021; Drake et al., 2021; Elser et al., 2021). Prior studies among this population have examined the role of food and housing insecurity and other social determinants on HIV care engagement (Dominguez-Dominguez et al., 2024; Dsouza et al., 2024; Wilson et al., 2018). However, missing from the literature is an exploration of how the COVID-19 pandemic exacerbated social and economic disparities, which worsened many of these social determinants of health. Moreover, missing from the literature is a methodical examination of the intersection of race, sexuality, and COVID-19 mitigation policies on the lives of BLSMM. Thus, unambiguously, further studies are needed to thoroughly examine the impact of pandemic-related mitigation efforts, such as SIP orders, on BLSMM’s ability to provide for life’s basic necessities (i.e., food and shelter) and maintain optimal mental and physical health. To that end, the purpose of this qualitative interpretive phenomenological study was to understand the impact of COVID-19 SIP orders and resource insecurity on the mental and physical health and general well-being of BLSMM in California and New York during and after the pandemic. Our study was guided by two research questions. Generally, what are the lived experiences of BLSMM during the COVID-19 pandemic? Specifically, how did the SIP orders impact BLSMM’s resource security, health, and general well-being?

## Methods

### Research methodology and design

Qualitative research is a process of naturalistic inquiry that seeks an in-depth understanding of social phenomena within their natural setting (Sandelowski, 2004; Streubert-Speziale & Carpenter, 2003). Qualitative research’s primary focus is on the “why” rather than the “what” of any particular social phenomenon (Marshall & Rossman, 2006; Patton, 2002; Sandelowski, 2004). Qualitative research relies on the direct experiences of research participants and recognizes them as meaning-making agents in their everyday lives (Lincoln & Guba, 1985; Streubert-Speziale & Carpenter, 2003). Additionally, qualitative research seeks to uplift marginalized and oppressed voices and empower people to share their unique narratives (Thambinathan & Kinsella, 2021; Zavala, 2013). Interpretive phenomenology, a qualitative methodology (Ray, 1994), was employed to explore and understand BLSMM experiences with COVID-19 SIP orders, its impact on resource security, and how these experiences impacted their health and well-being during and after the pandemic. Phenomenology is an attempt to approach a lived experience with a sense of “newness” to elicit rich and descriptive data (Streubert-Speziale & Carpenter, 2003). Bracketing is a process of setting aside one’s beliefs, feelings, and perceptions to be more open or faithful to the phenomenon (Streubert-Speziale & Carpenter, 2003). In addition, our conceptual model, the socioecological model (SEM), which was first posited by Bronfenbrenner in 1979, along with an intersectional framework, rooted in Black feminist traditions (Bronfenbrenner, 2005; Collins, 2000; Crenshaw, 1991), supported our use of interpretive phenomenology to explore these complexities around BLSMM experiences. The SEM posits that human experiences and behaviors are influenced by not only their individual behaviors but also by forces of influence operating outside their control (i.e., interpersonal relationships, community, institution, political) (Golden & Earp, 2012). We utilized an intersectionality framework to complement the SEM. Intersectionality is a critical theoretical framework rooted in Black feminist activism and scholarship that illuminates how power and privilege are differentially structured for sociodemographic groups and provides a guide through which researchers can understand experiences of marginalization, oppression, and discrimination (Bowleg, 2012; Crenshaw, 1991; Else-Quest & Hyde, 2016). To that end, the nature of the pandemic, disproportionate COVID-19 cases, and the limited ability to SIP or adhere to public health quarantine measures highlighted the disparate impact of the pandemic on communities of color, specifically BLSMM.

### Procedures

The institutional review boards of both the University of California—San Francisco in San Francisco, California (IRB #21–34350, Reference #318589) and the University of Rochester in Rochester, New York (Study ID: STUDY00007264) approved all procedures for this study prior to the start of data collection. The study was limited to BLSMM in California and New York. Advertisements for the study were sent to local Black and Latino community organizations that provide services to the community in these two states. Advertisements were also posted on internet-based message boards (i.e., Facebook, Instagram, and Twitter) of members of the study team. Purposeful snowball sampling procedures were utilized, which allowed for us to maximize recruitment efforts as well as to allow for the selection of information-rich cases that were relevant to the phenomenon under investigation (Carlsen & Glenton, 2011; Sandelowski, 1995). Participants were required to affirm that they were (a) 18 years of age or older, (b) assigned male sex at birth, (c) live in California or New York at the time of the study, and (d) had anal or oral sex with a man within the last year. The study team used electronic and telephone communication to invite potential participants to respond voluntarily to an online demographic and behavior survey, which was administered via *Qualtrics (*a web-based platform*)*. Once eligible participants have successfully completed the screening process, the research team explained the study to each participant and answered any questions they may have had before beginning the consenting process. Individuals were provided with an electronic informed consent form before proceeding with the online survey. A member of the study team explained the content of the informed consent form and obtained verbal consent. All study participants were interviewed via Zoom, a web conferencing platform. All study interviews were audio-recorded and then transcribed verbatim. Participant interviews lasted between 75 to 90 minutes. Participants received a $50 electronic gift card upon completion of all study procedures.

### Participants

In total, 44 individuals between the ages of 19 to 65 years of age (*M* = 42) participated in this study. While all participants were assigned male sex at birth, some participants identified as male (*n* = 41), female (*n* = 1), or nonbinary (*n* = 2). Participants also identified as gay (*n* = 27), bisexual (*n* = 5), heterosexual (*n* = 1), and queer (*n* = 3). There were several participants who did not record a response for their sexual orientation or reported “other” (*n* = 8). Study participants were either from California (*n* = 23) or New York (*n* = 21). Racially, participants identified themselves as Black or of African heritage (*n* = 32) or Latino (*n* = 11). One person did not list their race. Levels of education varied across the sample, with the majority of the sample having an undergraduate (*n* = 11) or graduate education (*n* = 17). Employment status was also mixed with half the sample reporting employment (*n* = 21) with the remaining either unemployed or listed other (*n* = 19). A total of four persons did not list their employment status. While 44 individuals completed the demographic and behavior survey, only 41 individuals participated in the individual interviews. All efforts were exhausted to reach those four individuals. See Table 1 for other participant demographics.

### Research team positionality

The research team included ten members: five nurse practitioner master’s students, one medical student, a public health trained research coordinator, and three doctoral-prepared researchers. The lead researcher and first author of this manuscript identifies as an Afro-Caribbean, cisgender man, with extensive clinical and research experience working with sexual and gender minorities in the United States and the Caribbean. His own clinical and lived experiences underscored his interest in initiating this study. Moreover, the lead author’s positionality, a fusion of his collective identities, supported him in navigating and negotiating the insider/outsider perspective, which was also useful in building rapport with study participants (Anthias, 2002; Rose, 1997; Sultana, 2007). Additional researchers included those who identify as gay, bisexual, heterosexual, persons of trans experience, cisgender, genderqueer, and nonbinary. The study team also consisted of those who identify as African American, Afro-Caribbean, White, Latino, or of Middle Eastern descent.

Throughout the conduct of the study, research team members met frequently to reflect on and examine our individual positionality, subjectivities, or other experiences that may affect the collection and interpretation of the data (Creswell et al., 2007). Team members experiences include (a) an awareness and knowledge of issues affecting BLSMM and other sexual and gender minorities, in addition to other contextual and historical events pre-during-and post pandemic; (b) firsthand experiences as Black or Latino sexual or gender minority; and (c) collective experiences around the events related to the pandemic, the racial justice movement, and accessing sexual health services. As part of the reflexive approach, our team met frequently to debrief, bracket, and examine our biases during the data collection and analysis phases of the study (Creswell et al., 2007; Streubert-Speziale & Carpenter, 2003). In addition, a key feature of these meetings was centered around supporting team members as many of us represent these communities and were experiencing many of the events presented in this study. The lead and last author both provided a supportive space for research team members to reflect, communicate, and share their experiences.

### Data sources

#### Demographic questionnaire:

Each participant received a personalized invitation to complete a brief demographic and behavior survey. The survey included questions on educational level, race/ethnicity, income, sexual orientation and gender identity, sexual behaviors, relationship history, and employment status. The survey was administered via *Qualtrics* and took approximately 15–20 minutes to complete.

#### Semi-structured interviews:

The semi-structured interview guide was informed by the relevant literature on BLSMM experiences around accessing HIV prevention and care services, mental health, and social support services. Since the events of the pandemic and the SIP orders implemented to curtail the spread of COVID-19 were new, sections of the interview guide were created after consultation with key stakeholders from the community. The first author drafted a set of interview questions that the research team reviewed. The first author subsequently modified the interview guide throughout the study, based on the team’s feedback, and as the events around the COVID-19 pandemic evolved. The semi-structured interview guide includes topics such as: 1) impact on resource security; 2) healthcare systems utilization during and after the pandemic; 3) exposure to violence; 4) Shelter-in-place orders (SIP); and 5) Implementation of mask mandates. Some of the interview questions include: 1) What was your experience when the shelter-in-place order was implemented in 2020? 2) What were your thoughts or feelings about wearing a mask to combat COVID-19 during the pandemic? 3) Was there ever a point during the pandemic when you experienced housing instability? 4) Was there ever a point during the pandemic when you experienced disruption in your employment status? 5) Was there ever a point during the pandemic when you experienced a disruption in your ability to purchase groceries? And 6) What are some of the challenges you experienced while accessing health services during the pandemic? Although the guide was created prior to the interviews to allow for systematic sequencing of the content, flexibility facilitated deviations to less sensitive topics (Wimpenny et al., 2000).

#### Field notes:

Field notes were generated after the completion of each individual interview. Additionally, reflexive memos from members of the research team were all documented throughout the study and aided in the analysis of the data. The purpose of the field notes was to record the observations and impressions from study participants. Each field note and reflexive memos were either audio recorded or written in a personal journal owned by a member of the research team or the qualitative data management software. Field notes were limited to descriptions of the social and geographical context.

### Data collection and analysis

In-depth, individual, semi-structured interviews were the primary source of data collection (Guest et al., 2006; Wimpenny et al., 2000). Based on the existing literature and with consultation with key stakeholders in the community, an in-depth interview guide, informed by our theoretical and intersectional frameworks, consisting of open-ended questions was developed to cover several specific topic areas, which also offered consistency throughout the interviews (Wimpenny et al., 2000). Although the interview guide was developed to provide a systematic sequencing of the study topic areas, members of the research team were flexible and allowed for digressions around (sometimes more sensitive) topics, which provided variation in the conversations (Gill et al., 2008). All interviews were conducted in English.

Qualitative data management and analysis were conducted using the qualitative analysis computer software ATLAS.ti (Version 23.4). All in-depth interviews were audio-recorded and immediately transcribed by a University of California, San Francisco IRB-approved transcriptionist who is trained in confidentiality and human subject protection. All de-identified transcripts were then uploaded to and managed using ATLAS.ti (Web and desktop versions). An interpretive phenomenological approach to thematic analysis and a collaborative coding process was employed to identify themes (Lopez & Willis, 2004). All team members were trained by the lead author, an expert in qualitative research, on qualitative research design and analysis before the start of the data collection process. A hallmark of the analysis process in interpretive phenomenological research is to evaluate the data to identify a paradigm case (i.e., strong instance of a pattern of action that hangs together) or exemplar narratives (i.e., smaller narratives that highlight, extend, add nuance, and variability to patterns observed) that illustrates the phenomenon under investigation (Pacherie, 2008; Ray, 1994). In addition, another component of interpretive phenomenological studies is the process of horizontalization. In this study, horizontalization was performed by providing equal value and importance to each of the narratives and coding them with descriptive labels (Lopez & Willis, 2004; MacKey, 2005).

**Figure 1.**
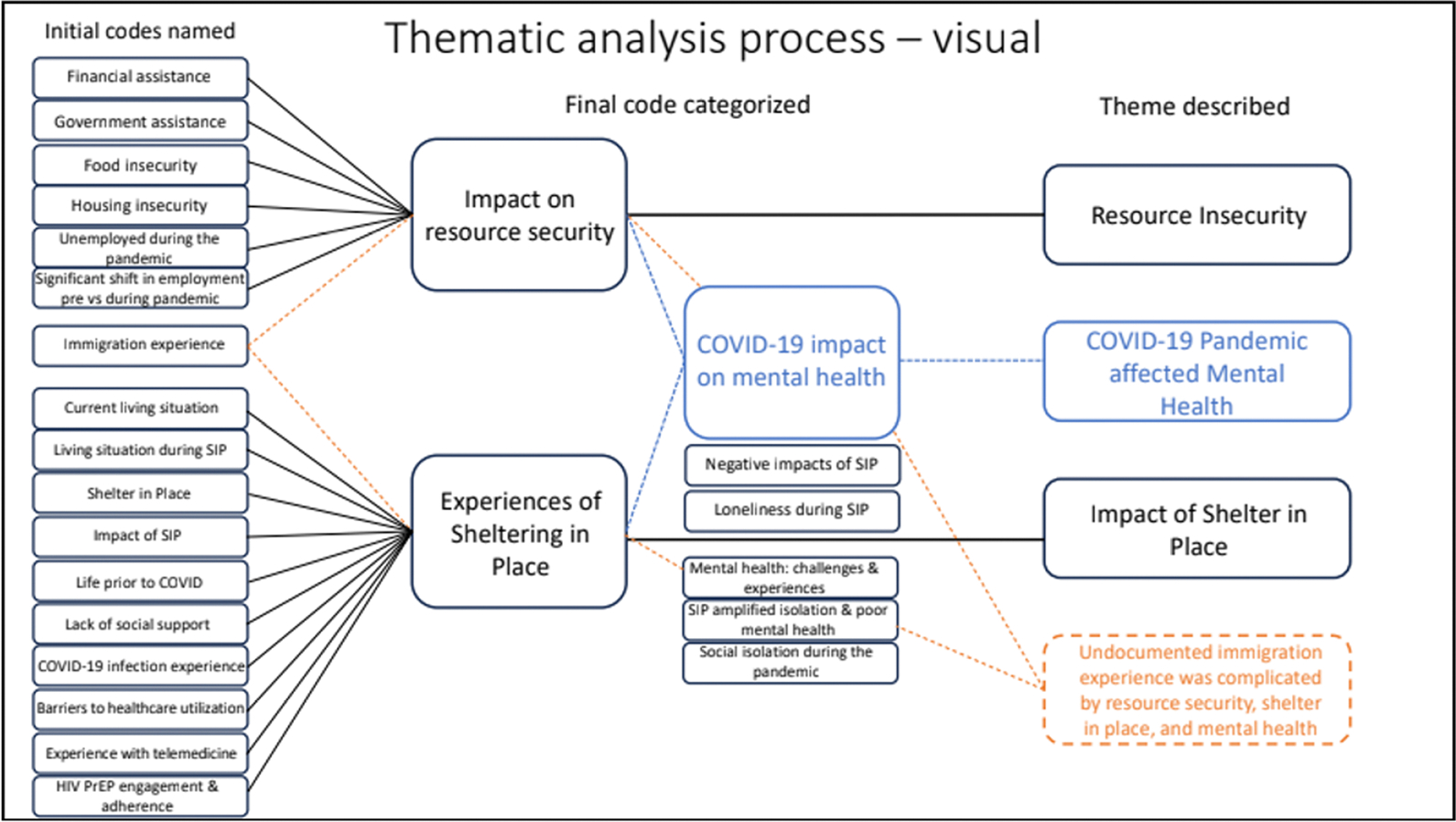
Intersectional thematic analysis process for understanding the impact of COVID-19 shelter-in-place orders and resource insecurity on BLSMM health and well-being.

Data analysis was executed in a stepwise fashion by the research team consisting of the principal investigator, co-investigator, project manager and four research assistants. Although codes were not created *a prior*, each transcript was reviewed and coded by members of the research team using an open coding technique to capture large passages of meaningful text, which continued throughout the analysis process (Braun & Clarke, 2006). Initial codes (*n* = 143) derived from the opening coding process were reviewed, compared with each other for similarities, and ended with either the collapsing of related or the elimination of duplicate codes, yielding a final codebook consisting of 81 codes. The next step in the data analysis process was to review the narratives attached to a code and produce detailed summary statements or “definitions” for each code. Code summaries were then reviewed and evaluated by the entire team for further commonality and alignment. Codes with similar topic areas were clustered together and later represented as categories (Patton, 2002). For example, codes labeled “financial assistance,” “government assistance,” “food insecurity,” “housing insecurity,” and “unemployed during the pandemic” were clustered together into a single category labeled “impact on resource security.” Each category was evaluated for similarities, which led to further refinement. Data analysis continued until saturation was achieved (Patton, 2002). Saturation was achieved when no new knowledge or themes emerged, which indicates that further data collection and analysis were necessary. The final step in the analytical process concluded with the development of themes. Themes were generated by reviewing categories with respect to a given research question. Upon reflection on the question, the team collectively evaluated code categories to identify one or more common themes or subthemes across categories. Once themes were identified across categories, thematic statements were written to reflect the common theme(s) across different categories. See Figure 1 for a visual representation of the thematic analysis process guiding the results around the impact of COVID-19 shelter-in-place orders and resource insecurity in BLSMM health and wellbeing.

### Methodological integrity

We employed several strategies in the data analysis process in order to ensure methodological integrity and trustworthiness. First, we engaged with the data by reviewing each transcript and audio for accuracy. All inaccuracies were reviewed and corrected with consensus from research team members before continuing with the coding of the data. Second, we employed investigator triangulations to further establish trustworthiness by having six members of the research team independently code the data and then meet as a group to discuss our findings (Breitmayer et al., 1993; Creswell et al., 2007). We utilized our weekly group meetings to address inconsistent findings between coders during a consensus-seeking process, with the goal of reaching full agreement among all coders; the first and last author led all debriefing meetings. Other forms of ensuring trustworthiness included bracketing assumptions and biases around BLSMM COVID-19 SIP experiences and resource insecurities by utilizing fieldnotes and memos, journaling (Creswell et al., 2007), and examining exemplar case examples using an intersectional lens by focusing on those participants whose experiences might be different or same. As an example, research team members believed that being undocumented and identifying as a racial/ethnic minority (and the intersection of those identities) would exacerbate the experience of resource insecurity for BLSMM with those marginalizations. To address this, we purposely evaluated the data during the analysis process to identify these and other differences.

## Results

### Demographic characteristics of study participants

Table 1 presents the sociodemographic characteristics of the sample. A total of 63 individuals were contacted for participation in this study. Of these individuals, 44 agreed to complete the study survey and participate in an individual interview. However, of these individuals, only 41 participated in one of the individual interviews with a member of the research team. The overall mean age of participants was 42 years (range 19–65), 73% Black/African American, Latino 25%; 64% had an undergraduate or graduate education; 93% identified as male; and 73% identified as gay or bisexual.

### Central themes

The data presented in this report is a direct result of the three themes and one subtheme: (a) impact of SIP; (b) resource insecurity; (c) COVID-19 pandemic affected mental health; and the subtheme—(d) undocumented immigration experience was complicated by resource security, SIP, and mental health. In the subsequent sections of the results section, we outlined the three themes and the subtheme and provided narrative exemplar quotes to illustrate our findings.

### Impact of shelter-in-place (SIP)

SIP was a socially and physically isolating period during the pandemic for many participants. Participants reported living alone or with roommates, parents, partners, relatives, or other friends during the pandemic. For most participants, proximity with members of their household led to increased irritation and arguments amongst their household members, roommates, or intimate partners during SIP. Those who were either undocumented or recently released from incarceration relied on shelters for housing during SIP. For those who identified as introverted, SIP was a period for them to avoid large crowds and interactions, which also helped those with chronic illnesses such as HIV to avoid potential exposure to COVID-19. Participants were selective with whom they interacted with during this time, leading to a reduction in their social networks. For those participants who were more extroverted, SIP negatively impacted them in terms of losing close connections with peers, resulting in them becoming more reclusive. For example, one participant states, *“I am not a homebody, and I hate being in the house unless it’s taking a nap or sleep or do the normal things. But I will say it was mentally very hard, very hard for me.”* The fear of the unknown with the COVID-19 virus and the implementation of SIP orders for some dramatically changed their social environment and culture, resulting in an increase in television and alcohol consumption.

I understand how connected I was to other humans at the time. I was a very extroverted kind of person, and I really became very reclusive in those first couple of months, because I did not know how to respond to being on a college campus where there were no employees on the campus, there were no students on the campus. [X State], to give you perspective, is in a village with one stoplight. So, there aren’t many people in general, and the college is kind of what makes the population. [Black, New York, 29 years old]

Participants’ access to healthcare during the pandemic was complicated by their fears of contracting COVID-19, lack of health insurance, and uncertainty around what impact COVID-19 might pose to their immune systems for those living with HIV. COVID-19 contact fears included not wanting to risk exposure in their provider’s office, the need to rely on public transportation, unvaccinated patients, or simply just walking around. Some participants reported an extended period, some around 2 years, without accessing healthcare services, extending beyond the onset of the pandemic. Others who had appointments scheduled to see their providers (primary care, dentist, ophthalmometry) in 2020 reported having all those appointments canceled due to the pandemic and SIP orders.

Definitely access to being face to face. That was a little bit like learning curve for me. It’s like okay, I can’t be seen. Or just a fear of like contracting the virus if I go to be seen. I remember one time I had to get, I had to go in because I needed new glasses and stuff and I needed to have a new exam. It was quite the process as far as the COVID protocols. Going through that and also the fear of getting COVID. [Black, California, 38 years old]

SIP orders also disrupted engagement in HIV prevention health services. For example, some participants stopped taking HIV pre-exposure prophylaxis (PrEP; medication to prevent HIV) because the pandemic interrupted sexual activity, leading to long periods of sexual inactivity. While those who were sexually active reported that exposure to an STI during the pandemic was a reason to continue or to start PrEP. For those participants who were not on PrEP, the beginning of the pandemic and the implementation of the SIP orders impacted their ability to initiate and maintain PrEP access during the pandemic. Some participants commented that their appointments were canceled in early 2020 as the pandemic was beginning to take hold in the United States, and many did not follow up to reschedule those appointments.

I went a year without health insurance, and for me that was the gap for me when it came to PrEP. I had it in school, I had health insurance in school, but navigating, just getting a job, a lot of jobs just kind of pushed to Medi-Cal, and then trying to figure out that space and finding a provider, my mind wasn’t even on PrEP, unfortunately. But it did affect my lifestyle as well. The same type of risk management, like I don’t want to put myself in a situation when I know I don’t have the resources. [Black, California, 24 years old]

### Resource insecurity

Resource insecurity has been a recurring phenomenon for many participants. For many participants, their experience with resource insecurity started prior to the pandemic and goes as far back as early as childhood. Some participants came from families that relied on government assistance programs for food and housing. These early experiences provided some with the knowledge and strategies they needed to employ in order to access these programs during the pandemic. During the COVID-19 pandemic, resource insecurity manifested in many ways, requiring participants to rely on a number of different social programs or their individual social networks (i.e., family and friends) in order to provide for housing, food, and other necessities. Financial assistance from family and friends involved support around paying bills, buying food, or providing a place to stay. Participants expressed receiving government assistance in the form of supplemental nutrition assistance program (SNAP) benefits, social security insurance (SSI), electronic benefit transfers (EBT), disability benefits, cash assistance, and COVID-19 relief payments. One example of such benefits was reflected in this participant’s narrative, “*I’m unemployed. I am receiving SNAP benefits, and I have a few scholarships.*” Others, if they were in school, relied on refunds from student loans or scholarships.

There was a point, because I had savings, I did put off considering filing for unemployment. We didn’t want to, we agreed not to apply for it because we knew that it could potentially have negative impacts on like our tax returns and stuff like that, and we didn’t want to complicate that in any way... Eventually it did get to a point where I did try applying, and I don’t know if I did it wrong or I don’t know what happened, but I ended up not qualifying for whatever reason. [Black, California, 32 years old]

Right now, I’m blessed because I have a Section 8 voucher. So, I’m able to keep [my apartment]. If it wasn’t for my Section 8, I couldn’t afford to have an apartment. It’s too expensive. I only get $952 a month. That’ll be on rent, and then this year in the summertime, right now I’m barely covering all my expenses. But in the summer when the electricity goes up and you need air conditioning and all that, I’m kind of worried about that because I don’t know how I’m going to pay the bill. I don’t get enough money. I’m going to have to go back to work or try to make some money somehow. [Latino, California, 55 years old]

During the pandemic, participants experienced significant shifts in their employment. Many participants worked in service, construction, education, and other essential industries that required them to continue work during COVID-19, while some worked in nonessential industries—which led to them losing their jobs. Those who worked in nonessential industries lost their jobs due to budget cuts and SIP orders (shutdowns). Therefore, many participants were unemployed for long periods of time, some throughout the entire pandemic. The period of unemployment induced fear of financial hardship for some and provided an opportunity for others to explore other interests and support others during this time. The pandemic allowed them time to process where they were currently in their lives and what changes they needed to make for self-improvement or change in their careers. Significant shifts in employment during the pandemic meant that those who were still able to work saw their jobs going remote—leaving some to lament about losing track of time or having days bleeding into each other.

When COVID-19 barely started and before I got diagnosed [with HIV], and before I started working with [X community organization] in 2020, I was basically unemployed due to services, and everything being shut down. And before [X community organization], I didn’t know how to basically get into this field and do these types of things. So that was the only time that I’ve been unemployed. [Black, California, 22 years old]

I was working in construction and a restaurant. Construction was Monday, Wednesday and Friday. The restaurant was five days a week. I was a dishwasher, part-time. When COVID-19 hit, our restaurant closed down and the construction people lost their contracts, so they got rid of people. I filed for unemployment and had to wait a year. I got my back pay, and I haven’t been back to work since. I’m still unemployed right now. I live off my back pay. I’m still living off that right now. I did like side jobs, like moving furniture or some side hustles. Maybe a little prostitution here and there, not so frequent, but I managed. [Black, New York, 40 years old]

Housing and Food insecurity emerged as two main factors contributing to resource insecurity during the COVID-19 pandemic. Housing insecurity was viewed by study participants as a double-edged sword—where some expressed not being exactly unhoused, and others expressed not being exactly housed. Participants who were dependent on university housing at the time of the pandemic reported being displaced and moving back home to stay with their parents or other relatives. For other participants, their housing insecurity was a direct result of their substance use history, stringent governmental criteria to qualify for housing assistance programs, history of incarceration, or due to immigration status. Those who qualify for assistance alleviated housing insecurity with housing assistance programs (for those living with HIV), unemployment insurance or benefits, or social support networks (i.e., family and friends).

As soon as I got out my parole officer didn’t have any housing or anything for me. They had switched, I guess, parole officers at the last minute and there was a lack of communication between the state prison and parole and all that. Then when I got out, I ended up walking to a homeless shelter called ‘The [X Center]’ that someone in jail told me about. It was a 90-day shelter, and it just so happened they had one opening, and I got in there for two months. They had a case worker who hooked me up with [X] HDAP program, so after two months, I got into housing through the HDAP program. A lot of people couldn’t find housing, or they couldn’t get into shelters. I mean, I just happened to luck out. The shelter where I was at, shortly before I got there wasn’t even taking new people. They were full, but because of the shelter-in-place orders, they had people there who were there for a year due to COVID lockdown and everything. They had just reopened right before I got released [from prison]. They were starting to transition people out into housing. So, I was fortunate. [Latino, California, 60 years old]

Food insecurity was another resource insecurity experienced by study participants during the pandemic. Most participants reported having pre-exposure to food insecurity as many experienced this during childhood. Participants reported having limited access to nutritious foods due to unemployment during the pandemic, financial instability, and substance use disorder. Participants were able to manage their experiences with food insecurity by rationing food, relying on friends or family, accessing food pantries and other charitable community organizations (i.e., Salvation Army, Catholic Charities, GMAC, Cal-Fresh), government food programs such as food stamps, or transition programs (post-incarceration). Those who did not express relying on any social programs acknowledged that “*food is expensive*.” These experiences triggered a visceral emotional response where some expressed disbelief that things had gotten so dire.

Just when I was, just like recently, in between jobs. So, looking for a job but still working, or still driving for Uber and that. So, like I mentioned, buying those things that are very inexpensive such as like ramen noodles. Luckily, it’s just me so it’s not as hard to buy food and make it last, I should say. So, just making things kind of like stretching out resources. [Black, California, 38 years old]

### COVID-19 pandemic and SIP orders affected mental health

A recurring theme that emerged from participants’ narratives was the feeling of isolation and the lack of social interactions with friends, family, co-workers, and romantic partners. For some participants, this experience led to increased anxiety, sadness, and depression, causing them to fluctuate between panic and peace, feeling like the world was going to end, anxious about grocery stores running out of items, and worried or afraid about how the world would recover from everything shutting down. The feeling of isolation was more profound for those participants who reported that they lived alone during the pandemic. Isolation from their loved ones and social groups for many participants increased their feelings of sadness, depression, and anxiety.

It was simple things, really. Just like for a time going to, leaving my house and going to the store or just like hanging out with friends. I was very lonely for a time, so that was a big factor in mental health and things. I would say the hardest to deal with was, I would say definitely the, for a time the curfew that was first put into place, I believe it was like 8:00 or something like that. So, I think that was, on top of not being able to, only going to the store to get what you needed, on top of that I would say the curfew. [Black, California, 38 years old]

...at first there was no effect on like my mental health, but again, once we started going into like the second year and the isolation really started setting in, it did amplify my depression a bit because I didn’t have the friends and the coworkers and the classmates and all those people anymore. So that isolation really got amplified and it wasn’t great. [Black, California, 38 years old]

While some viewed that period of isolation as depressing, others reported finding solitude in the experience. Participants who lived alone at home during that period of SIP reported that their pets, often a dog, made the experience more tolerable. For example, one participant states *“I do live alone, but I have a dog, so I’m like, she lives here too. But yeah, so it was just kind of like me and my dog. And I was alone.”*

Participants also experienced significant changes in their daily routine including unemployment, moving to remote work, losing access to social outlets (gym, bars, public spaces, etc.), being around household members consistently throughout the day, and quitting school due to transition to online learning. Some share that being unemployed and having excessive free time was very difficult. Others share working from home resulted in unclear boundaries between work and leisure time. Many participants reported negative self-image due to increased weight gain during SIP. With the closure of gyms, a common place for socialization for gay and bisexual men, many reported not having the ability to engage in physical activity. Others reported making time for physical activities outside of the gym space—including hiking, swimming, workout at home, or just taking care of their body by other means.

I was definitely one of those people who gained weight because of the pandemic. Not leaving the house anymore, not going to campus anymore, so I was walking significantly less. I was still exercising, but leaving the house and commuting adds so much physical activity that just disappeared. [Black, California, 32 years old]

I couldn’t go to the gym. I hated that, because I do work out frequently, five to six days a week. Couldn’t work out, had to work out at home, didn’t like that. Couldn’t go out to clubs. I still, I could do turn up, so I couldn’t go out to the clubs, that was not big fun. [Black, New York, 36 years old]

The use of virtual technology platforms that were supposed to enhance connection (i.e., Zoom and Facetime) was perceived by some to alleviate some feelings of loneliness and isolation. However, some found the persistent reliance on technology as overwhelming and burdensome, causing more anxiety and longing for in-person human interaction. It also requires access to resources and strong motivation to organize opportunities for connection. The effects of the SIP orders on mental health continue in the immediate post-pandemic era, modifying the way several participants socialize (i.e., who they talk to and the way they talk to people and requiring a longer warming up period), with some preferring to talk virtually over being in-person.

Most of my family, my aunts, and my mother are all over the age of 60 and living, I’m living far away from them trying to make sure that they’re okay, they have the most appropriate information. But then, yeah, at the end of my business day, I didn’t have anyone to talk to. Yes, I had my friends, but all of us were in a very similar situation. But you can’t see anybody, you couldn’t be in contact. The Zoom platforms were new, so you’re trying to do Zoom dinners or happy hours or whatever, but you still don’t get that same feeling of closeness. [Black, New York, 52 years old]

### Undocumented immigration experience

Undocumented immigration experience emerged as a subtheme in the analysis of the data, which cuts across all three major themes. While all participants experienced resource insecurity, mental health challenges, and SIP order difficulties, those who reported their undocumented immigration status reported significant challenges. In this study, we did not explicitly inquire about participants’ immigration status; however, several participants disclosed that they were undocumented. They also expressed that their being undocumented meant that they did not qualify for any of the government pandemic-related relief benefits. Participants’ immigration experience was emblematic of the structural racism that exists in U.S. immigration law. For one participant, his immigration challenges did not start during the pandemic but prior to. Participants noted that because of legislation signed into law in 1996, they were ineligible for any governmental assistance during the COVID-19 pandemic (housing, food, or financial assistance).

I had a stipend job at the time. I was in a program, the [X] transitional housing program, because I’m not eligible being undocumented, stateless. I’m not eligible thanks to Bill Clinton’s 1996 immigration law. We’re not eligible for housing or vouchers or any financial assistance or anything like that... [Black, New York, 51 years old]

Lucky for me, I am a documented immigrant as of now, right? It’s so easy to become undocumented or be deported. It was harsh, but it was definitely worth it. The homelessness I experienced was better than what I had experienced before I came here [United States]. I immigrated in 2019... I mean, it’s extremely expensive, but I am proud to have achieved a lot in my life with hard work and a lot of luck. And I’m very afraid to lose everything, and it’s possible because my status is not secure... I was literally living in a shelter, and didn’t have a work permit, so I couldn’t work. [Latino, New York, 24 years old]

Another participant spoke about his experience immigrating to the United States as a child and not being documented, which led to periods of homelessness, food insecurity, incarceration, and the persistent fear of deportation. For this participant, as well as several others in the study, the intersection of being Black or Latino, gay, male, and undocumented meant that they were in a perpetual state of survival. Undocumented participants reported feeling a lack of safety and security, which impacted their general well-being.

I know that these [resources] weren’t targeted at people like me with all of my intersectional issues. It focused more on the LGBTQ part... not the person of color or the Black man part. There are also the issues of being undocumented, being stateless, not being able to resolve, or being legally precluded from resolving your immigration issues. I am not homeless, but I also don’t have my own apartment. So, it’s like a semi-unhoused type of situation. I had to go through a whole lot of logistics and stuff to have a space for myself. [Black, New York, 51 years old]

## Discussion

A significant contribution of this paper is the illumination and exploration of the experience of BLSMM navigating COVID-19 shelter-in-place orders (SIP), the resulting resource insecurity, and when combined, both impacted their health and well-being. The findings from this study provided in-depth context-rich information about how the COVID-19 pandemic impacted this population, an area that has been significantly understudied. Our findings revealed three major themes and one subtheme: (a) impact of SIP, (b) resource insecurity, (c) COVID-19 pandemic affecting mental health, and the subtheme (d) undocumented immigration experiences complicated resource security, SIP, and mental health. Our methodological approach, informed by our application of both the socioecological and intersectional frameworks, aided in our ability to understand how COVID-19 quarantine measures, which resulted in unemployment for many participants and resource insecurity (e.g., being unstable or unhoused and food insecure), resulted in BLSMM having limited or no access to healthcare during the pandemic.

The participants in our study reported that COVID-19 mitigation measures contributed to them experiencing social and physical isolation that lasted for an extended period of time. The COVID-19 mitigation measures impacted participants differently based on whether or not they had considered themselves to be introverted or extroverted. The introverts in our study found these measures, specifically the ones dealing with social distancing, to be helpful in avoiding large crowds, which could increase the spread of COVID-19. On the other hand, for those who were extroverted, social distancing and SIP recommendations negatively impacted their ability to maintain close connections with friends. Whether participants considered themselves introverted or extroverted, social technologies can be a useful tool to counter the negative effects of SIP and other COVID-19 mitigation measures. Several studies have suggested that SIP and other mitigation measures resulted in an increase in depression, anxiety, sexual risk behaviors, and contributed to loneliness among SMM (Gabster et al., 2022; Holloway et al., 2021; Levy et al., 2022). Another consequence of SIP was the significant weight gain participants experienced due to physical inactivity. This finding is not unique to the BLSSM in this study, as similar results have been observed quantitatively in other populations (Carriedo et al., 2020; Goitia et al., 2022). However, our findings provide a nuanced qualitative perspective.

A significant experience of study participants was the impact of the pandemic and the subsequent SIP orders on their ability to access and engage in vital HIV prevention services. Some participants reported that many of their preventative medical appointments, including appointments for PrEP medications, were canceled at the start of the pandemic. As a result of those cancellations, many participants discontinued taking PrEP, stopped testing for STIs, or declined to follow up with their providers to reschedule appointments. However, for some participants, exposure to an STI during the pandemic was reported as a facilitator to restarting PrEP or re-engaging with the healthcare system but few were able to access a healthcare provider. As a harm reduction strategy in the absence of PrEP or access to STI testing, some participants changed their sexual behaviors, a strategy that was common among other SMM during this time in similar settings (Daroya et al., 2023). In addition, there were other factors that made accessing healthcare difficult during the pandemic for participants, such as the loss of health insurance, fear of contracting COVID-19, and not wanting to risk exposure to the virus from their provider’s office or from using public transportation. Although our participants came from two different states, New York and California, we found no difference in their experiences as it pertains to their access to HIV prevention services. While there were no observable differences, it is important to note that each state had its own SIP orders, with some being more liberal than others, underscoring the need for flexible public health policies and pandemic mitigation strategies instead of a “one-size-fits-all” approach (Feyman et al., 2020).

SIP orders in both New York and California constrained resources for food, housing, and employment for most of the participants in our study. In order to overcome many of these challenges, some participants relied on government assistance, unemployment benefits, family, a partner, or friends to provide for basic necessities. While the experience of resource insecurity was not a new phenomenon for most participants, several of whom reflected on early childhood exposure to parents accessing government assistance programs, their collective narratives suggested that these experiences brought about mixed emotions as they accessed these resources as adults. Apart from having limited access to food, many of our participants experienced significant shifts in their employment as those in nonessential industries were made redundant from their jobs or required to work limited hours if they were in essential industries. Social distancing and other pandemic mitigation guidelines were cited as the primary reason for many people within the US being made redundant or transitioning to remote work (Drake et al., 2021; Hagen et al., 2022). However, transitioning to report work for some participants was seen as a privilege as many of them worked in service industries where in-person attendance was necessary (Berry et al., 2021; Elser et al., 2021). In addition to food insecurity and unemployment, housing insecurity also emerged as another factor contributing to resource insecurity during the pandemic. The participants in our study viewed housing insecurity as a double-edged sword, with some suggesting that they were not exactly housed or unhoused. Government assistance or support from family members such as parents or friends aided in alleviating instances of housing insecurity for many participants as they provided a place for them to stay. The experiences of these men reflected the larger experiences of most people in the US during the pandemic (Couch et al., 2020). However, our findings are compelling when considered in the context of structural racial barriers and inequities exposed during the COVID-19 pandemic and how these structures disproportionately impacted communities of color, especially those who were employed as essential workers (Couch et al., 2020; Grekousis et al., 2022). These findings also support the assertion that publicly funded assistance programs have the potential to lower the impact of food and housing insecurity as well as unemployment (Baggett & Gaeta, 2021; Drake et al., 2021; Molitor et al., 2021).

The participants in our study reported that their mental health was significantly impacted due to the pandemic and the resulting SIP orders implemented in their jurisdiction. They reported feelings of isolation and lack of social interaction with family, friends, and romantic partners as factors contributing to the negative impact on their mental health. In addition, SIP orders also resulted in increased symptoms of anxiety, sadness, and depression. Many of these feelings occurred during the height of the pandemic when a lot of mental health services were either paused or went remote via telemedicine. Most of our participants reported not having access to mental health services or not having privacy at home to engage with a therapist because they lived in a multigenerational household. These two barriers to mental health services also emerged in the literature on SIP orders and social distancing guidelines. For example, one study with LGBTQ youth found that being isolated with unsupportive families and loss of in-person identity-based socialization and support were all factors contributing to poor mental health among study participants (Fish et al., 2020). While loneliness and isolation have impacted many people during the pandemic, most of the studies that have been done during this period have primarily focused on older adults (Krendl & Perry, 2021), with limited studies focusing on how these issues affected young or marginalized and minoritized adults, such as the men in our study. Our findings begin to shed light on how the pandemic and SIP orders have affected the participants in our study and provide an opportunity for more research among this population.

The COVID-19 pandemic and the SIP orders in both New York and California affected some participants more severely because of their immigration status. Undocumented immigration experience emerged from the analysis as a subtheme because it was echoed across all three of the major themes—the impact of SIP, resource insecurity, and COVID-19 affecting mental health. While we did not actively probe for immigration status, nor was it an eligibility criterion, it emerged as a significant barrier to participants’ being able to navigate the challenges of the pandemic and SIP. For example, being undocumented meant that some participants were unable to access publicly funded government assistance programs or any of the pandemic relief benefits. In addition, the syndemic impact of homelessness, food insecurity, incarceration, and the looming fear of deportation all contributed to poor mental health among participants who were undocumented. Taking in context, these findings mirrored anti-immigrant sentiments within the society and propagated by the former president of the United States, especially around undocumented individuals becoming a public charge (Hearst et al., 2021). Additionally, the intersection of being Black/African American or Latino as well as a sexual and gender minority and undocumented exacerbated the additive impact of discrimination and racism during a time of hypervigilance because of COVID-19 and the police murder of George Floyd (Hearst et al., 2021; Page et al., 2020; Sönmez et al., 2020).

The application of both the social-ecological model and intersectionality as a framework to better understand COVID-19 SIP orders and resource insecurity experiences provided an opportunity to understand these unique experiences among BLSMM, a subpopulation that is stigmatized and marginalized (i.e., racism, homophobia, HIV stigma, undocumented immigrant). The intersectionality framework and the SEM model offered a lens through which we were able to identify and examine the overlapping impact of social identities (i.e., race, sexual orientation, immigration status, and socioeconomic status), social inequalities, and other structural factors (i.e., housing insecurity, food insecurity, and access to healthcare) on BLSMM experience the COVID-19 pandemic. Intersectionality framework, rooted in Black feminist pedagogy and advocacy, guided our analysis and explained how the COVID-19 mitigation measures, such as the SIP orders, resource insecurity, mental health challenges, and being undocumented, exacerbated social and economic disparities among the BLSMM in our study. Our findings are similar to that of the emerging literature on how the COVID-19 pandemic disproportionately affected minoritized and marginalized groups (Bhuiyan et al., 2021; Drake et al., 2021; Lodge & Kuchukhidze, 2020; Mackey et al., 2021; Parra et al., 2023). An interesting observation made in this study is that educational attainment, with a majority (63%) of participants reporting having an undergraduate education or higher and almost half being employed (48%), did not increase BLSMM resource security or improve general well-being. This observation, along with additional evidence from the literature, suggested that race, undocumented status, and other social determinants influenced resource security, mental and physical health, and overall well-being (Baggett & Gaeta, 2021; Millett et al., 2020; Page et al., 2020; Webb Hooper et al., 2020).

Our findings have several implications. These include advocacy around the implementation of equitable policies that center the needs of communities of color who are more likely to experience profound health and economic disparities. In addition, there are several opportunities for future research that employs an intersectional, social justice, and health equity lens to better understand the disproportionate impact of the COVID-19 pandemic or future pandemics on the lives of BLSMM, especially those who are undocumented. First, we found that SIP orders and other COVID-19 mitigation policies during the pandemic disparately impacted the health and overall well-being of many of the participants in our study. SIP orders have been proven to increase rates of depression, anxiety, loneliness, domestic and sexual violence, and hazardous sexual behaviors (Berry et al., 2021; Feyman et al., 2020; Leslie & Wilson, 2020; Weber et al., 2021). Many of the SIP orders that were implemented were a one-size-fits-all approach. Our findings suggest a more nuanced and culturally appropriate approach that is tailored to the unique needs of the community in that local jurisdiction. For example, increased access to affordable housing to reduce the reliance on multi-generational housing. Second, the pandemic and SIP orders across these two states disrupted our participants’ ability to access vital HIV prevention services, with many of them having their PrEP appointments canceled at the beginning of the pandemic or discontinuing taking Pre-exposure prophylaxis (PrEP) due to lack of access. This suggests the need for healthcare providers to make telemedicine accessible and develop and implement alternative PrEP delivery and STI testing programs to improve access to care for this population. Third, resource insecurity appeared to have affected participants’ ability to afford housing or food, increasing their reliance on government assistance programs. During the pandemic, the unemployment rate for the Black and Latino populations was higher than the national average, underscoring the need for publicly funded social protection programs that are accessible to all, including those with mixed immigration status. To achieve this, for example, the government can lower the poverty rate in order to make more people eligible for government food, health, or housing assistance programs. In addition, the government can also remove or suspend policies that prohibit undocumented individuals from being able to access public assistance during a public health crisis.

## Limitations

The present study has several limitations. First, our participants were predominantly from two urban areas along the western and eastern coast of the United States; therefore, the experiences of those BLSMM who live in rural or southern parts of the country were unrepresented in the study. Second, the overall findings in this interpretive phenomenological qualitative study are limited to the small sample of individuals who participated in the study. Although study participants are not representative of the experiences of all racialized sexual minority men, the perceptions and experiences shared in this study have relevance and applicability beyond the men who participated in this study. Third, while we did have age diversity among study participants, those with diverse gender identities were less represented, which is due in part to the focus of the study. Fourth, not all participants who were contacted participated in the study. Also, three participants completed the demographic and behavior survey but chose not to participate in the individual interviews. Fifth, our sample consisted primarily of individuals who were college-educated. The perspectives of those who are less educated were underrepresented in this study and are in need of further investigation. Sixth, the study sample consisted mostly of BSMM, with LSMM comprising less than 30% of the sample; therefore, the narratives and experiences of LSMM may not be fully understood. Finally, our study interviews occurred a year after the onset of the pandemic, which meant that some areas would have lifted their SIP orders. This may have impacted our participants’ responses to the interview questions. Therefore, with these limitations, we assert that our interpretations of the findings in this study are limited to this sample of Black and Latino MSM living in both California and New York.

## Conclusion

While the imminent threats of the COVID-19 pandemic have waned and SIP orders implemented to curb the spread of the virus have concluded nationally, there is a renewed focus and attention on the various disparities (both health and economic) that developed within the US that disproportionately affected communities of color. BLSMM in the United States, especially those living with or at risk for acquiring HIV, continue to experience health and economic disparities that exacerbate disease progression, decrease quality of life, and result in poor mental health. Our research, which was informed by our application of the socioecological model and intersectional frameworks, helped to improve our understanding of how the COVID-19 pandemic and shelter-in-place orders intensified the experience of food and housing insecurity, unemployment, and lack of access to healthcare—specifically, vital HIV prevention and treatment services among BLSMM. The findings suggest that publicly-funded social assistance programs, such as the ones mentioned in this study, have the potential to lower resource insecurities during a time of increased economic hardships. Finally, efforts to decrease or eliminate disparities in health and to increase access to food and shelter must be in partnership with local community organizations that provide services to communities highly impacted.

## Figures and Tables

**Table 1.** Sociodemographic Characteristics of the Sample (*N* = 44)

	*n*	%

Age (*mean= 42.43 years)*		
19–29 years	9	20.5
31–39 years	12	27.2
40–49 years	11	25.0
51–56 years	6	13.7
60–65 years	5	11.3
Missing	1	2.3
Sex assigned at birth		
Male	44	100.0
Gender		
Male	41	93.2
Female	1	2.3
Non-binary/third gender	2	4.5
Sexual orientation		
Gay	27	61.4
Bisexual	5	11.4
Heterosexual or straight	1	2.3
Queer	3	6.8
Other^a^	4	9.1
Missing	4	9.1
Race/Ethnicity		
Black or African American	32	72.7
Hispanic or Latinx	11	25.0
Missing	1	2.3
Site		
California	23	52.3
New York	21	47.7
Levels of education completed		
High School	8	18.2
Junior College or Vocational School	3	6.8
Undergraduate School	11	25.0
Graduate or Professional School	17	38.6
Other^b^	1	2.3
Missing	4	9.1
Employment Situation		
Unemployed	9	20.5
Employed	21	47.7
Other^c^	10	22.7
Missing	4	9.1

a Other: Pan, Aego-, and Demisexual; Demisexual; Same-Gender Loving.

b Other: GED.

c Other: Retirement; SSA; General Relief and Food Stamps; Internship; SSDI; SSI; Student.
